# Cadmium Exposure and Risk of Breast Cancer by Histological and Tumor Receptor Subtype in White Caucasian Women: A Hospital-Based Case-Control Study

**DOI:** 10.3390/ijms20123029

**Published:** 2019-06-21

**Authors:** Loreta Strumylaite, Rima Kregzdyte, Algirdas Bogusevicius, Lina Poskiene, Dale Baranauskiene, Darius Pranys

**Affiliations:** 1 Neuroscience Institute, Lithuanian University of Health Sciences, Eiveniu 4, LT-50161 Kaunas, Lithuania; rima.kregzdyte@lsmuni.lt (R.K.), dale.baranauskiene2@lsmuni.lt (D.B.); 2 Department of Surgery, Medical Academy, Lithuanian University of Health Sciences, Eiveniu 2, LT-50009 Kaunas, Lithuania; algirdas.bogusevicius@kaunoklinikos.lt; 3 Department of Pathological Anatomy, Medical Academy, Lithuanian University of Health Sciences, Eiveniu 2, LT-50009 Kaunas, Lithuania; lina.poskiene@lsmuni.lt (L.P.); patkmuk@kmu.lt (D.P.)

**Keywords:** urinary cadmium, breast cancer, tumor receptors, case-control study

## Abstract

As the majority of experimental studies suggest cadmium being metalloestrogen, we examined cadmium/breast cancer (BC) association by histological and tumor receptor subtype in 509 invasive BC patients and 1170 controls. Urinary cadmium was determined by atomic absorption spectrometry, and categorized using tertiles of its distribution in the controls: <0.18, 0.18–0.33, >0.33 kg × 10^−9^/kg × 10^−3^ creatinine. Relative to the lowest category of urinary cadmium adjusted odds ratio (OR) of ductal BC was 1.18 (95% confidence interval (CI): 0.89–1.58) in the intermediate and 1.53 (95% CI: 1.15–2.04) in the highest category. There was a significant association for hormone receptor-positive ductal BC: ORs per category increase were 1.34 (95% CI: 1.14–1.59) for estrogen receptor-positive (ER+), 1.33 (95% CI: 1.09–1.61) for progesterone receptor-positive (PR+) and 1.35 (95% CI: 1.11–1.65) for ER+/PR+ BC. We found a significant association between cadmium and human epidermal growth factor receptor 2-negative (HER2−) ductal BC. The strongest association with cadmium was for ER+/PR+/HER2− ductal BC. The associations between cadmium and lobular BC with hormone receptor-positive and HER2− were positive but insignificant. There was no evidence that the associations with cadmium differed for cancers with different tumor histology (*p*-heterogeneity > 0.05). This study provides evidence that urinary cadmium is associated with the risk of hormone receptor-positive and HER2− breast cancer independent of tumor histology.

## 1. Introduction

Cadmium is one of the most toxic metals that is present in the environment naturally and due to human activities in industry and agriculture. In the general population, the main sources of cadmium are cigarette smoke, food, water and ambient air, especially, in urban areas or nearby industrial settings [1]. Whereas, in the non-smoking population, food items such as cereals, vegetables and potatoes are the main source of cadmium [2]. In the respiratory and gastrointestinal tracts absorbed cadmium has a long half-life in the human body and accumulates in the kidneys. Cadmium concentration in urine is in close correlation with cadmium levels in the body burden and in the kidneys [1,2]. Therefore, urinary cadmium concentration is assessed as a long-term exposure biomarker [1]. 

Emerging evidence suggests that cadmium is an endocrine-disrupting chemical that demonstrates estrogen like activity. Most experimental studies address the ability of cadmium to activate genomic and non-genomic pathways of estrogen receptor (ER)-α [3]. In vitro studies show that cadmium induces the proliferation of estrogen dependent breast cancer cells [4,5,6,7], activates ER-α in transfection assays [4,5,6,8], increases the transcription and expression of genes, such as the progesterone receptor (PR), that are regulated by estrogen [4,9]. Cadmium also increases signaling through the extracellular signal-regulated kinases 1/2 and Akt pathways [9,10,11]. Studies in vivo demonstrate cadmium mimicking the effects of estradiol in the mammary gland and/or the uterus, and inducing expression of PR and complement component 3 [12,13,14]. The ability of cadmium to mimic estrogen effects in the mammary gland may contribute to breast cancer development. It has been found that cadmium effects can also be mediated by the ER independently from estradiol [4]. However, some studies failed to show estrogen like activity of the metal [15] or demonstrated other action pathways [16]. 

Several epidemiological studies have shown that a higher cadmium concentration in urine was associated with an increased risk of breast cancer [17,18,19,20], but not all [21,22]. Previously we reported a greater risk of breast cancer for certain tumor receptor (ER+, human epidermal growth factor receptor 2 (HER2)−) subtypes in relation to urinary cadmium [23]. However, we found only one epidemiological study that evaluated how cadmium is associated with the risk of different histological types of breast cancer. Ductal carcinoma accounts for 40–75% and lobular carcinoma for 5–15% of all invasive breast carcinomas [24]. In addition to differences in their histopathology, lobular carcinomas are more often hormone receptor-positive compared with ductal carcinomas [25]. Because of mentioned disparities and, possibly, different risk factors for breast cancer histological subtypes, in this article we explore the association between cadmium concentration in urine and invasive breast cancer defined by tumor histology and receptor status.

## 2. Results

Invasive breast cancer patients (cases) and controls were Caucasian, similar with respect to age and marital status. Family history of breast cancer, age at menarche and first birth, menopausal status, smoking, and body mass index also did not differ between cases and controls (Table 1). However, cases were more educated, with a longer estrogen-active (fertile) period, gave birth fewer times, more frequently used alcohol and hormone therapy during menopause, and had endocrine diseases (diabetes mellitus and thyroid diseases) more often diagnosed. Urinary cadmium concentration was also higher in cases than in controls. 

From multivariable adjusted logistic regression, increased concentration of urinary cadmium was associated with a significant increase in the odds of invasive breast cancer: The OR per category increase in cadmium was 1.24 (95% CI: 1.09–1.42) (*p*-trend = 0.002) (Table 2). Stratifying the cases by hormone receptor, the association appeared significant only among hormone receptor-positive (ER+, PR+, ER+/PR+) breast cancer: The OR per category increase in urinary cadmium was 1.34 (95% CI: 1.14–1.56) (*p*-trend < 0.001) for ER+, 1.36 (95% CI: 1.13–1.63) (*p*-trend = 0.001) for PR+ and 1.37 (95% CI: 1.14–1.65) (*p*-trend = 0.001) for ER+/PR+ breast cancer (Table 2). However, there was no significant heterogeneity in the associations comparing ER+ and ER− (*p* = 0.11), PR+ and PR− (*p* = 0.18) or ER+/PR+ and ER−/PR− breast cancer (*p* = 0.09). 

A higher level of cadmium in urine was associated with an increased risk of HER2− but not HER2+ breast cancer: A 1 category increase in urinary cadmium was associated with an OR of 1.3 (95% CI: 1.12–1.50) (*p*-trend < 0.001) for HER2- breast cancer and 0.99 (95% CI: 0.73–1.35) (*p*-trend = 0.97) for HER2+ breast cancer (*p*-heterogeneity = 0.12) (Table 2). When stratified by joint tumor receptor status, there was a significant positive association between urinary cadmium and ER+/PR+/HER2− breast cancer: The OR per category increase was 1.43 (95% CI: 1.18–1.73) (*p*-trend < 0.001), with a suggestion that this differed from the urinary cadmium and ER−/PR−/HER2+ breast cancer association (*p*-heterogeneity = 0.05) (Table 2).

Because of different tumor receptor status in ductal and lobular carcinomas, we explored associations between urinary cadmium and risk of ductal and lobular tumors restricting the analysis to ER+, PR+, ER+/PR+, HER2− and ER+/PR+/HER2− breast cancer (Table 3). Both ductal and lobular carcinomas with selected tumor receptor status demonstrated positive associations with urinary cadmium; however, significant associations were found only among patients with ductal carcinoma. Relative to the lowest category of urinary cadmium, adjusted OR of ductal breast cancer was 1.18 (95% CI: 0.89–1.58) in the intermediate and 1.53 (95% CI: 1.15–2.04) in the highest category (*p*-trend = 0.003). There was a significant association for hormone receptor-positive ductal breast cancer: ORs per category increase were 1.34 (95% CI: 1.14–1.59) for ER+, 1.33 (95% CI: 1.09–1.61) for PR+ and 1.35 (95% CI: 1.11–1.65) for ER+/PR+ breast cancer. We also found a significant association between cadmium and HER2− ductal breast cancer: The OR per category increase was 1.30 (95% CI: 1.11–1.51). However, the strongest association with cadmium was for ER+/PR+/HER2− ductal breast cancer: The OR per category increase was 1.41 (95% CI: 1.15–1.74). The associations between cadmium and lobular cancer with hormone receptor-positive and HER2− were positive but insignificant. There was no evidence that the associations with cadmium differed for cancers with different tumor histology (*p*-heterogeneity > 0.05).

## 3. Discussion

In this study, we identified a positive association between urinary cadmium concentration, which is a known long-term exposure biomarker, and risk of invasive breast cancer. White Caucasian women with urinary cadmium > 0.33 kg × 10^−9^/kg × 10^−3^ creatinine had a 1.55-fold increase in the odds of invasive breast cancer compared with women with cadmium <0.18 kg × 10^−9^/kg × 10^−3^. Other case-control studies showed a 2–3-fold increased risk of breast cancer for women with the highest concentration of cadmium in urine [17,18]. The strongest cadmium/breast cancer association was reported in a Japanese case-control study: Women with urine cadmium > 2.6 kg × 10^−9^/kg × 10^−3^ had 6 times higher risk compared to the subjects with cadmium in urine < 1.674 kg × 10^−9^/kg × 10^−3^ [19]. Some authors reported 16% increased risk of breast cancer for every 1 kg × 10^−9^ of cadmium increase per 1 kg × 10^−3^ of creatinine [20]. A meta-analysis including five case-control studies and one cross-sectional study suggested that higher urinary cadmium levels were related to an increased risk of breast cancer [26,27] with pooled ORs of 2.24 for the highest vs. lowest cadmium category and 1.66 for each 0.5 kg × 10^−9^ of cadmium increase per 1 kg × 10^−3^ of creatinine [26]. However, two case-cohort studies found little evidence of an association between urinary cadmium levels and breast cancer [21,22]. This may be due to several reasons. First, in both studies cadmium was measured in baseline urine samples and may differ considerably from cadmium measured at the time of diagnosis/enrollment in the subcohort, because of the ability of cadmium to accumulate in the human body, and likely changes in diet and smoking (the main cadmium sources in the general population) over time, especially, when case-cohort study within a Women’s Health Initiative study included individuals from clinical trials involved in modification of diet (a low-fat eating pattern) [21,28]. Second, the time from baseline to diagnosis/enrollment may differ between the cases and subcohort, because the cases and subcohort members were not matched on this variable. Third, one of these studies did not require that the urine sample be taken in the morning [22], unlike the majority of previous studies.

Most experimental studies corroborate cadmium/hormone receptor-positive associations providing evidence on estrogenicity of cadmium and its ability to activate the genomic and non-genomic pathways of ER-α [3,4,5,6,7,8,9,10,11]. In this study, we identified significant urinary cadmium/invasive breast cancer associations for hormone receptor-positive (ER+, PR+ and ER+/PR+), but not for hormone receptor-negative (ER−, PR− and ER−/PR−) breast cancer subtypes. However, heterogeneity in the associations between cadmium and either receptor-positive or receptor-negative breast cancer was not significant. One of the reasons could be the limited number of patients with certain breast cancer subtypes. Another reason could be related to measurements of ER and, consequently, the use of ER but not ER-α in this and other epidemiological studies. It can partly explain some variations between the results obtained in different studies.

We found that cadmium was associated with HER2−, but not HER2+ invasive breast cancer. This finding is, in part, supported by the experimental study reported ability of cadmium to cause malignant transformation of normal human breast epithelial cells into a basal-like cancer phenotype with the negativity of the HER2 and ER-α, decreased susceptibility of breast cancer gene 1, and increased expression of cytokeratin 5 and p63 [16]. However, after stratification of the cases by joint receptor status, we did not find an association of cadmium with ER−/PR−/HER2− breast cancer. This could be due to taking into account limited criteria for a basal-like cancer phenotype, and the small number of cases. 

The strongest association was with ER+/PR+/HER2− invasive breast cancer, with a suggestion that this association is different from that with ER−/PR−/HER2+ breast cancer (*p*-heterogeneity = 0.05). These results are partly in agreement with experimental studies supporting estrogen like activity of cadmium via different pathways of ER-α [3].

We found no evidence of heterogeneity in the associations between urinary cadmium and invasive ductal or invasive lobular carcinomas of the corresponding tumor receptor subtypes. However, significant risk related to cadmium concentration in urine was found only for invasive ductal ER+, PR+, ER+/PR+, HER2− and ER+/PR+/HER2− breast cancer. Hormone receptor-positive, HER2− and ER+/PR+/HER2− lobular breast cancer was also sensitive to cadmium, but due to the limited number of cases with invasive lobular carcinoma in the study group the relationships were not statistically significant. Other authors also did not find any difference in cadmium related risk of breast cancer by tumor histology [22].

We used unconditional logistic regression (adjusted for age and other risk factors) to estimate associations. We did not include any food items in the model, to avoid underestimation of the cadmium/breast cancer association, since urinary cadmium reflects cumulative exposure from all possible sources [1]. However, smoking was included in the model because of other possible carcinogens in tobacco.

One of the strengths of this study is that exposure to cadmium was assessed by urinary cadmium, which is proportional to the body burden and used as a long-term exposure biomarker in epidemiological studies [1]. By measuring urinary cadmium in the same laboratory under the same conditions and using the same quality standards for both cases and controls, we avoided one of the greatest limitations of case-control studies related to recall biases in exposure assessment. 

Another strength is the use of only newly diagnosed invasive breast cancer patients within the study period with no other cancer diagnosed in the past. This made it possible to avoid some effects on cadmium level in urine because of changes in lifestyle and previously used treatment (surgery, chemotherapy, radiation, etc.). Nonetheless, 6.5% of cases had preoperative chemotherapy before surgery, and 1.8% of cases provided urine 2–3 days after surgery. However, this is unlikely to have a strong influence on our results. McElroy et al. [17] reported a significant relationship between urinary cadmium and breast cancer risk in a group of cases who had experienced surgery and/or radiation.

A potential limitation of the study is the use of hospital-based controls, who may not be representative of the general population. However, in this study, the proportion of single individuals, higher educated individuals, and daily smokers among controls aged 45–64 years (50% of all controls) was similar to that in the target population [29].

Another limitation of the study is related to recall biases common to case-control studies. However, using hospital-based controls may be an advantage, because all women had an illness, which meant the controls were more likely to report information on potential risk factors in a similar way to the cases.

We measured only ER, not ER-α, which is a target exploring the mechanism of cadmium action. This could partly explain some inconsistency of epidemiological findings with the results obtained in experimental studies.

A potential limitation was the relatively small number of patients with lobular carcinoma and, consequently, limited number of cases in some strata defined by tumor receptor status. Therefore, some of our estimates of association had wide confidence intervals, limiting the possibility to draw firm conclusions.

In spite of that, we observed a urinary cadmium/invasive breast cancer association that was not explained by bias, confounding or chance. However, some uncertainty whether this is a causal relationship or a consequence of the disease process per se remains. Therefore in vitro and in vivo studies using cadmium as a causal agent are very useful. The majority of experimental studies corroborate the ability of cadmium to activate pathways of ER-α and provide evidence of its estrogenicity [3,4,5,6,7,8,9,10,11].

## 4. Materials and Methods 

### 4.1. Study Design

A hospital-based case-control study of breast cancer risk factors was performed between 1 March 2007 and 10 January 2011 in the Hospital of Lithuanian University of Health Sciences Kauno Klinikos. The cases (*n* = 585, response rate 86.9%) were women aged 28–90 years with new histologically confirmed breast cancer (C50 (malignant neoplasm of breast) and D05 (carcinoma in situ of breast) according to the International Classification of Diseases (ICD) 10th Revision), free from other cancer diagnosed in the past and undergoing surgical treatment at the Department of Surgery. In our histology specific analyses, we included 509 invasive breast cancer patients with histology in medical records classified as ductal (*n* = 458, ICD for Oncology (ICD-O) code 8500) or lobular (*n* = 51, ICD-O code 8520). The 76 cancer patients with other ICD-O histology codes were excluded from the analyses. The controls (*n* = 1170, response rate 84.1%) were women free from any cancer undergoing treatment for a wide spectrum of non-neoplastic disorders and diseases of breast, eye, ear-nose-throat, nervous and cardiovascular systems in various departments of the hospital. We received written informed consent from each individual before they participated in the study. The study was conducted in accordance with the Declaration of Helsinki. The study protocol was approved by the Kaunas Regional Biomedical Research Ethics Committee (10-01-2007 No. BE-2-1, Report No. 5/2007).

### 4.2. Questionnaire

Both invasive breast cancer patients and controls were asked to complete a questionnaire, which was validated and reliable for collection of data on demographic and socioeconomic characteristics, personal history of diseases, height and weight, family history of cancer, woman’s reproductive history, and lifestyle (smoking, use of alcohol, diet, and etc.) [30]. 

### 4.3. Urine Sample Collection

We collected urine samples from cases and controls on 1–2 days after admission to the hospital. We gave oral instructions on how to obtain urine samples to both cases and controls, asking for approximately 1–3 × 10^−5^ m^3^ of first morning urine in a special plastic container, after hygiene of perineum. Plastic tubes with the samples of urine (5 × 10^−6^ m^3^) were stored at +5 °C until analysis.

### 4.4. Cadmium Concentration Measurements

We used atomic absorption spectrometry (Perkin-Elmer, Zeeman 3030, USA) to determine cadmium concentration in urine [31]. A simultaneous analysis of a standard reference urine (Seronorm^TM^, REF 201305) was carried out to ensure the accuracy and precision of the analytical procedures for cadmium concentration in urine. In the reference urine, the certified value for cadmium was 0.31 × 10^−9^ kg/m^3^ × 10^−3^. Analyzing the reference urine (*n* = 36), we got mean and standard deviation (SD), and the coefficient of variation values of 0.32 ± 0.03 × 10^−9^ kg/m^3^ × 10^−3^ and 10.2%, respectively. We used low sorption plastic tubes and labware prepared according to previously described protocol [32].

### 4.5. Measurements of ER, PR, HER2 and Creatinine

We measured the ER, PR and HER2 levels in the specimens of breast tumor tissue using immunohistochemistry [33]. According to the results, obtained invasive breast cancer patients were hormone receptor-positive or negative [34]; HER2+ with value of 3+; and HER2− with values of 0 and 1+ [35]. The cases with HER2 value of 2+ were checked additionally by in situ hybridization and were assigned to HER2+ or HER2− according to the result. 

To control kidney function [36], we measured creatinine in urine by the Jaffe method [37]. To assess possibly different effects of kidney on cadmium level in urine, we expressed urinary cadmium levels as urine cadmium/urine creatinine (kg × 10^−9^/kg × 10^−3^ creatinine).

### 4.6. Statistical Analysis

We summarized baseline characteristics of invasive breast cancer cases and controls computing means and SD for continuous variables and frequencies for categorical variables. Unpaired t-tests for continuous variables and chi-squared tests for categorical variables were used comparing characteristics between cases and controls. For variables (creatinine-adjusted urinary cadmium level, urinary creatinine) with skewed distributions, we calculated median and 25 and 75 percentiles. To compare creatinine-adjusted urine cadmium and creatinine between cases and controls, we used the Wilcoxon rank-sum test. 

According to tumor histology, we stratified the cases to ductal and lobular cancer patients; according to tumor receptor status the cases were stratified as follows: ER+, ER−, PR+, PR−, ER+/PR+, ER+/PR−, ER−/PR+, and ER−/PR−; HER2+, and HER2−. We also grouped the cases into 8 categories defined by joint tumor receptor status: ER+/PR+/HER2−, ER+/PR+/HER2+, ER−/PR−/HER2−, ER−/PR−/HER2+, ER+/PR−/HER2−, ER+/PR−/HER2+, ER−/PR+/HER2−, and ER−/PR+/HER2+. However, because of insufficient statistical power, we excluded cases with ER−/PR+ (*n* = 13), ER+/PR+/HER2+ (*n* = 12), ER+/PR−/HER2+ (*n* = 9), ER−/PR+/HER2+ (*n* = 2), and ER−/PR+/HER2− (*n* = 11) breast cancer. 

We used unconditional logistic regression to estimate the association between urinary cadmium and breast cancer subtypes, calculating odds ratios (ORs) and 95% confidence intervals (CI). We adjusted the model for possible risk factors of breast cancer such as age, number of births, age at first birth, estrogen-active (fertile) period, hormone therapy during menopause (never, estrogens and/or estrogens-progestin, other), family history of breast cancer in first and/or second degree of relatives (no, yes, unknown), use of alcohol (never or ex-, current), smoking (never, ex-, current (every day or sometimes)), body mass index, education (specialized secondary or lower, some university or higher), marital status (single, married or living as married, separated or widowed), diabetes mellitus (absent, present), and thyroid diseases (absent, present). Cadmium in urine was categorized using tertiles of its distribution in the controls. Heterogeneity in urine cadmium and each breast cancer subtype associations was tested by a Cochran Q-test. The level of statistical significance was set at 0.05. All reported P-values are 2-sided. To perform analyses, we used Stata 10 [38]. 

## 5. Conclusions

In spite of some limitations of the study, it provides evidence of an association between urinary cadmium, as a biomarker of lifetime exposure to cadmium, and risk of invasive breast cancer, dependent on tumor hormone receptor status, but not histology. A greater risk of invasive breast cancer for patients with hormone receptor-positive and human epidermal growth factor receptor 2-negative cancer in relation to increased urinary cadmium is corroborated by experimental studies supporting several mechanisms of cadmium action. However, further experimental and epidemiological studies that could define cadmium effect biomarkers, and take into account individual susceptibility, would be very useful to obtain more information on the potential causal relationship between cadmium and breast cancer. Since the main sources of cadmium are smoking (active and passive) and food, public health messages should be developed which advise women how to reduce their exposure to cadmium.

## Figures and Tables

**Table 1:** Characteristics of invasive breast cancer cases and controls.

Variable	Cases (*n* = 509)	Controls (*n* = 1170)	*p*-Value for Difference
**Age (years) (mean, SD)**	58.08 (12.32)	57.42 (12.49)	0.31
**Education (n, %)**			
Specialized secondary or lower	304 (59.7)	816 (69.7)	
Some university or higher	205 (40.3)	354 (30.3)	<0.001
**Marital status (n, %)**			
Single	30 (5.9)	55 (4.7)	
Married or living as married	308 (60.5)	712 (60.9)	
Separated or widowed	171 (33.6)	403 (34.4)	0.58
**Family history of breast cancer (n, %)**	33 (6.5)	58 (5.0)	0.2
**Age at menarche (years) (mean, SD)**	14.03 (1.72)	14.01 (1.69)	0.82
**Age at first birth (years) (n, %)**			
<20	70 (13.8)	190 (16.2)	
20–29	353 (69.3)	830 (70.9)	
≥30	35 (6.9)	65 (5.6)	
Never gave birth	51 (10.0)	85 (7.3)	0.12
**Number of births (mean, SD)**	1.77 (1.09)	1.92 (1.08)	0.01
**Menopausal status (n, %)**			
Premenopausal	156 (30.7)	347 (29.7)	
Postmenopausal	353 (69.3)	823 (70.3)	0.68
**Estrogen-active (fertile) period (years) (mean, SD) ^a^**	34.35 (5.97)	33.21 (6.34)	<0.001
**Hormone therapy during menopause (n, %)**			
Never	285 (80.7)	725 (88.1)	
Estrogens and/or estrogens-progestin	47 (13.3)	71 (8.6)	
Other hormones (thyroxin and etc.)	21 (6.0)	27 (3.3)	0.004
**Alcohol use (n, %)**			
Never/Ex-user	72 (14.2)	263 (22.5)	
Current	437 (85.8)	907 (77.5)	<0.001
**Smoking (n, %)**			
Never	386 (75.8)	930 (79.5)	
Ex-smokers	56 (11.0)	127 (10.8)	
Current (every day or sometimes)	67 (13.2)	113 (9.7)	0.1
**Body mass index (kg m** **^−2^) (mean, SD)**	28.11 (5.56)	28.54 (5.94)	0.16
**Diabetes mellitus (n, %)**	35 (6.9)	118 (10.1)	0.04
**Thyroid diseases (n, %)**	112 (22.0)	317 (27.1)	0.03
**Urinary cadmium (kg × 10^−9^/kg × 10^−3^ creatinine) (median,** Q_1_, Q_3_**)**	0.29 (0.18–0.43)	0.24 (0.15–0.40)	0.002
**Urinary creatinine (kg × 10^−9^/m^3^ × 10^−3^) (median, Q_1_, Q_3_)**	1.35 (0.90–1.99)	1.19 (0.83–1.75)	0.001
**Tumor histopathological characteristics**			
**Histology**			
Invasive ductal carcinoma	458 (78.3)	-	
Invasive lobular carcinoma	51 (8.7)	-	
**Receptors (n, %)**			
Estrogen receptor-positive	348 (68.4)	-	
Progesterone receptor-positive	243 (47.7)	-	
Human epidermal growth receptor 2-negative	435 (85.5)	-	

Abbreviations: SD, standard deviation; CI, confidence interval; Q_1_, 25 percentile; Q_3_, 75 percentile. ^a^ Estrogen-active (fertile) period (years) = current age for non-menopausal women or age at menopause for postmenopausal women (years) minus age at menarche (years). All variables are in bold.

**Table 2:** Odds ratios (ORs) and 95% confidence intervals (CIs) for the association of creatinine-adjusted urinary cadmium (kg × 10^−9^/kg × 10^−3^ creatinine) with breast cancer.

Controls/Cases	Number in Each Category of Urinary Cadmium (n, %) ^a^			OR ^b^ (95% CI)		OR ^b^ per Category	*p*-Trend	*p*-Heterogeneity *
	Low (<0.18)	Intermediate (0.18–0.33)	High (>0.33)	Intermediate vs. Low	High vs. Low	Increase (95% CI)		
**Controls**	389 (33.3)	392 (33.5)	389 (33.3)					
**Cases**	133 (26.1)	170 (33.4)	206 (40.5)	1.24 (0.94–1.63)	1.55 (1.18–2.04)	1.24 (1.09–1.42)	0.002	
ER+	82 (23.6)	114 (32.8)	152 (43.7)	1.30 (0.94–1.80)	1.79 (1.30–2.46)	1.34 (1.14–1.56)	<0.001	0.11 (1)
ER−	51 (31.7)	56 (34.8)	54 (33.5)	1.10 (0.72–1.67)	1.16 (0.75–1.79)	1.07 (0.87–1.33)	0.52	-
PR+	55 (22.6)	84 (34.6)	104 (42.8)	1.48 (1.01–2.15)	1.88 (1.30–2.74)	1.36 (1.13–1.63)	0.001	0.18 (2)
PR−	78 (29.3)	86 (32.3)	102 (38.4)	1.03 (0.72–1.45)	1.29 (0.91–1.82)	1.14 (0.96–1.35)	0.14	-
ER+/PR+	52 (22.6)	77 (33.5)	101 (43.9)	1.42 (0.96–2.09)	1.90 (1.30–2.78)	1.37 (1.14–1.65)	0.001	0.09 (3)
ER+/PR−	30 (25.4)	37 (31.4)	51 (43.2)	1.05 (0.62–1.75)	1.54 (0.94–2.54)	1.26 (0.98–1.62)	0.07	0.33 (4)
ER−/PR−	48 (32.4)	49 (33.1)	51 (34.5)	1.02 (0.66–1.58)	1.13 (0.73–1.76)	1.06 (0.85–1.33)	0.58	-
HER2+	28 (38.9)	17 (23.6)	27 (37.5)	0.58 (0.31–1.09)	0.99 (0.56–1.77)	0.99 (0.73–1.35)	0.97	0.12 (5)
HER2−	104 (23.9)	152 (34.9)	179 (41.2)	1.41 (1.05–1.89)	1.70 (1.27–2.29)	1.30 (1.12–1.50)	<0.001	-
ER+/PR+/HER2−	46 (21.1)	74 (33.9)	98 (45.0)	1.54 (1.03–2.30)	2.08 (1.41–3.09)	1.43 (1.18–1.73)	<0.001	0.05 (6)
ER+/PR−/HER2−	29 (26.6)	35 (32.1)	45 (41.3)	1.02 (0.60–1.74)	1.40 (0.84–2.34)	1.19 (0.92–1.54)	0.18	0.32 (7)
ER−/PR−/HER2−	28 (28.9)	36 (37.1)	33 (34.0)	1.30 (0.76–2.21)	1.26 (0.73–2.18)	1.12 (0.86–1.47)	0.40	0.48 (8)
ER−/PR−/HER2+	20 (40.0)	12 (24.0)	18 (36.0)	0.57 (0.27–1.20)	0.94 (0.47–1.86)	0.95 (0.66–1.37)	0.79	-

Abbreviations: ER, estrogen receptor; PR, progesterone receptor; HER2, human epidermal growth factor receptor 2. **^a^** Creatinine-adjusted urinary cadmium concentration (kg × 10^−9^/kg × 10^−3^ creatinine) categorized using tertiles of the distribution in the controls; low: < 0.18, intermediate: 0.18–0.33, high: > 0.33. **^b^** OR adjusted for age, number of births, age at first birth, estrogen-active (fertile) period, hormone therapy during menopause, family history on breast cancer, alcohol use, smoking, body mass index, education, marital status, diabetes mellitus, and thyroid diseases. * *p*-value from Cochran Q test of heterogeneity in the associations between cadmium (per category increase) and (1) ER+ or ER− breast cancer, (2) PR+ or PR− breast cancer, (3) ER+/PR+ or ER−/PR−, (4) ER+/PR− or ER−/PR−, (5) HER2+ or HER2− breast cancer, (6) ER+/PR+/HER2− or ER−/ PR−/HER2+, (7) ER+/PR−/HER2− or ER−/PR−/HER2+, (8) ER−/PR−/HER2− or ER−/PR−/HER2+. All variables are in bold.

**Table 3:** Odds ratios (ORs) and 95% confidence intervals (CIs) for the association of creatinine-adjusted urinary cadmium (kg × 10^−9^/kg × 10^−3^ creatinine) with invasive ductal and invasive lobular breast carcinoma.

Controls/Cases	Number in Each Category of Urinary Cadmium (n, %) ^a^			OR ^b^ (95% CI)		OR ^b^ per Category	*p*-Trend	*p*-Heterogeneity *
	Low (<0.18)	Intermediate (0.18–0.33)	High (>0.33)	Intermediate vs. Low	High vs. Low	Increase (95% CI)		
**Controls**	389 (33.3)	392 (33.5)	389 (33.3)					
**Ductal cases**	122 (26.6)	151 (33.0)	185 (40.4)	1.18 (0.89–1.58)	1.53 (1.15–2.04)	1.24 (1.08–1.43)	0.003	0.89
ER+	72 (24.0)	96 (32.0)	132 (44.0)	1.23 (0.87–1.74)	1.79 (1.27–2.50)	1.34 (1.14–1.59)	0.001	0.86
PR+	50 (23.9)	71 (34.0)	88 (42.1)	1.36 (0.91–2.03)	1.79 (1.21–2.66)	1.33 (1.09–1.61)	0.004	0.49
ER+/PR+	47 (23.9)	64 (32.5)	86 (43.6)	1.29 (0.85–1.95)	1.83 (1.22–2.73)	1.35 (1.11–1.65)	0.003	0.62
HER2−	93 (24.2)	133 (34.6)	158 (41.2)	1.36 (1.00–1.88)	1.70 (1.25–2.31)	1.30 (1.11–1.51)	0.001	0.93
ER+/PR+/HER2−	41 (22.2)	61 (33.0)	83 (44.9)	1.41 (0.91–2.16)	2.02 (1.33–3.07)	1.41 (1.15–1.74)	0.001	0.76
**Lobular cases**	11 (21.6)	19 (37.3)	21 (41.2)	1.72 (0.80–3.71)	1.73 (0.80–3.75)	1.27 (0.88–1.83)	0.19	-
ER+	10 (20.8)	18 (37.5)	20 (41.7)	1.77 (0.80–3.93)	1.80 (0.81–4.00)	1.29 (0.89–1.89)	0.19	-
PR+	5 (14.7)	13 (38.2)	16 (47.1)	2.70 (0.94–7.75)	2.99 (1.05–8.51)	1.58 (1.00–2.50)	0.05	-
ER+/PR+	5 (15.2)	13 (39.4)	15 (45.4)	2.70 (0.94–7.75)	2.79 (0.97–8.00)	1.53 (0.96–2.43)	0.07	-
HER2−	11 (21.6)	19 (37.2)	21 (41.2)	1.72 (0.80–3.71)	1.73 (0.80–3.75)	1.27 (0.88–1.83)	0.19	-
ER+/PR+/HER2−	5 (15.2)	13 (39.4)	15 (45.4)	2.70 (0.94–7.75)	2.79 (0.97–8.00)	1.53 (0.96–2.43)	0.07	-

Abbreviations: ER, estrogen receptor; PR, progesterone receptor; HER2, human epidermal growth factor receptor 2. **^a^** Creatinine-adjusted urinary cadmium concentration (kg × 10^−9^/kg × 10^−3^ creatinine) categorized using tertiles of the distribution in the controls; low: < 0.18, intermediate: 0.18–0.33, high: > 0.33. **^b^** OR adjusted for age, number of births, age at first birth, estrogen-active (fertile) period, hormone therapy during menopause, family history on breast cancer, alcohol use, smoking, body mass index, education, marital status, diabetes mellitus, and thyroid diseases. * *p*-value from Cochran Q test of heterogeneity in the associations between cadmium (per category increase) and ductal or lobular carcinomas with the same tumor receptors.
